# Systematic review of patient and caregivers’ satisfaction with telehealth videoconferencing as a mode of service delivery in managing patients’ health

**DOI:** 10.1371/journal.pone.0221848

**Published:** 2019-08-30

**Authors:** Joseph F. Orlando, Matthew Beard, Saravana Kumar

**Affiliations:** 1 Physiotherapy Department, Royal Adelaide Hospital, Adelaide, Australia; 2 School of Health Sciences, University of South Australia, Adelaide, Australia; Universiteit Twente, NETHERLANDS

## Abstract

Telehealth is an alternative method of delivering health care to people required to travel long distances for routine health care. The aim of this systematic review was to examine whether patients and their caregivers living in rural and remote areas are satisfied with telehealth videoconferencing as a mode of service delivery in managing their health. A protocol was registered with PROSPERO international prospective register of systematic reviews (#CRD42017083597) and conducted in line with the Preferred Reporting Items for Systematic Reviews and Meta-Analyses (PRISMA) statement. A systematic search of Ovid Medline, Embase, CINAHL, ProQuest Health Research Premium Collection, Joanna Briggs Institute and the Cochrane Library was conducted. Studies of people living in rural and remote areas who attended outpatient appointments for a health condition via videoconference were included if the studies measured patient and/or caregivers’ satisfaction with telehealth. Data on satisfaction was extracted and descriptively synthesised. Methodological quality of the included studies was assessed using a modified version of the McMaster Critical Review Forms for Quantitative or Qualitative Studies. Thirty-six studies of varying study design and quality met the inclusion criteria. The outcomes of satisfaction with telehealth were categorised into system experience, information sharing, consumer focus and overall satisfaction. There were high levels of satisfaction across all these dimensions. Despite these positive findings, the current evidence base lacks clarity in terms of how satisfaction is defined and measured. People living in rural and remote areas are generally satisfied with telehealth as a mode of service delivery as it may improve access to health care and avoid the inconvenience of travel.

## Introduction

People living in rural and remote areas travel long distances to access health care not readily available in their local communities. The inconvenience and cost of travel [1, 2], absenteeism from work and family [1, 3, 4] and dependence on caregivers for transport or childcare [1, 4] create barriers to access. Vulnerable groups are placed under additional hardship, including people from low socioeconomic backgrounds [5], indigenous communities [6, 7], children and the elderly [8], people with disability [9] and people with multiple co-morbidities [10].

Telehealth is an alternative mode of service delivery that enables people living in rural and remote areas to access health care within their local communities. Telehealth refers to remote service delivery utilising information and communication technologies such as telephone, videoconferencing, electronic messaging or digital monitoring to improve health outcomes [11]. With the improving internet and infrastructure, videoconferencing in particular has gained increasing prominence in the delivery of telehealth. It may be seen to retain the benefits of traditional face-to-face appointments through real-time visual cues important for rapport building, clinical observation, visual assessment and sharing of resources or education materials [12, 13]. This is possible because, unlike pre-recorded formats such as the use of facsimile, videoconferencing allows for bi-directional and synchronous real-time communication between two or more stakeholders. Delivering health care in local community or directly into one’s home via videoconferencing can address inequalities and potentially reduce pressure on health services dealing with the rise in chronic conditions [14]. Telehealth may be a viable alternative to face-to-face appointments [11, 13, 15, 16].

Patient satisfaction is important for telehealth to be a viable mode of service delivery. Satisfaction with health care is closely linked to improved patient engagement and treatment compliance for a spectrum of conditions in different clinical settings [17, 18]. There is a large body of literature examining patient satisfaction with telehealth, but these studies are limited by poor methodological quality as they are often pilot studies set in experimental and temporal contexts [19]. Two previous reviews of the literature have reported high levels of patient satisfaction with telehealth. For example, Hilgart *et al*. [20] found common factors of satisfaction with technology, education and information provided, communication and avoidance of patient travel. Similarly, Kruse *et al*. [21] identified common factors of patient satisfaction related to health outcomes, modality use and preference, low cost and communication. While these findings are relevant, these literature reviews are limited due to methodological concerns with regards to absence of formal critical appraisal of the included literature as well as publication bias, such that searching was limited to only two databases and there was no searching of the grey literature [20, 21].

Therefore, the purpose of this systematic review was to address the current knowledge gap on patient satisfaction with telehealth, in particular through the use of videoconferencing, using robust methodology and also include caregivers’ satisfaction with regards to this mode of service delivery. As patients’ experiences living with a health condition and accessing health care are shared by caregivers [22, 23], this systematic review explored both stakeholders’ perspectives with telehealth.

## Materials and methods

A protocol was registered with PROSPERO international prospective register of systematic reviews (#CRD42017083597). This review was conducted and reported in line with the Preferred Reporting Items for Systematic Reviews and Meta-Analyses (PRISMA) statement [24]. Please refer to S1 File.

### Search strategy

Six electronic databases were searched, including: Ovid Medline, Embase, CINAHL, ProQuest Health Research Premium Collection, Joanna Briggs Institute and the Cochrane Library. The following search terms were used with MESH headings as relevant: telehealth, exp telemedicine, exp teleconsult, exp remote consultation, exp videoconferencing, exp patient satisfaction, exp caregivers, exp rural health, exp rural population, exp rural health services (S2 File). The search was limited to humans, English language and published between January 2003 to December 2017. Grey literature searching was undertaken using a commonly available internet search engine (Google). Pearling of reference lists of included studies was performed to identify additional articles.

All search results were pooled and duplicates removed. Titles and abstracts were screened before analysing the full texts to determine their eligibility. The screening process was undertaken by two independent reviewers (JO and SK). Any disagreements were resolved and discussed with a third reviewer (MB), where required.

### Study selection

All forms of prospective, primary research studies were considered. Secondary research, such as literature reviews, were excluded but their reference lists were searched to identify additional studies. Studies were included if patients and/or their caregivers living in rural and remote areas accessed health care for a clinical condition. Rural and remote included inner regional, outer regional, remote or very remote areas, but not metropolitan or urban areas. There was no exclusion of participant age. Studies were included if the intervention was delivered remotely using videoconferencing technology. While telehealth is not limited to videoconferencing, given the focus of this review was on videoconferencing, the term telehealth henceforth refers to videoconferencing. The telehealth intervention had to occur as part of an outpatient appointment between the patient in their home or local health care centre and a health care provider at another centre. Consultations from inpatient or emergency department were excluded as were use of videoconferencing for administrative purposes, such as scheduling of appointments. Patient and/or caregivers’ satisfaction utilising videoconferencing was the outcome of interest. Any quantitative and/or quantitative measure was included. Please refer to PICOT table in S2 File.

### Methodological quality assessment

A modified version of the McMaster Critical Appraisal Tools for Quantitative Studies [25] and Qualitative Studies [26] were used to assess the methodological quality of included studies depending on their study design. Quantitative studies were assessed over eight main components including: study purpose, literature review, study design, sample, outcomes, intervention, results and conclusions. The individual components were rated as ‘yes’, ‘no’, ‘not addressed’ or ‘not applicable’. A score of ‘1’ was given to ‘yes’, ‘0’ to ‘no’ and ‘not-addressed’ while items rated as ‘not applicable’ were omitted from the total score; the maximum total score being 14.

Qualitative studies were assessed over eight components including: study purpose, literature review, study design, sampling, data collection, data analysis, overall rigor and conclusions. The individual components were rated and scored as per quantitative studies; the maximum total score being 22. The methodological assessment of the included studies was undertaken by two independent reviewers (JO and SK). Any disagreements were resolved and discussed with a third reviewer (MB), where required. Studies were not excluded based on the methodological quality score.

### Data extraction

The data were extracted and collated into bespoke Microsoft Excel spreadsheets and the following domains of data were extracted from each study: study design; participants’ information (age, gender), sample size, country, clinical service, outcome measures and findings. Studies were categorised based on the dimensions of satisfaction results compared. Given the nature of the review question, a meta-analysis was not undertaken and instead a descriptive synthesis was used.

### Data synthesis

As means of interpretation of findings and implications for clinical practice, various dimensions of satisfaction were generated: system experience, information sharing, consumer focus and overall satisfaction. These dimensions were adapted from a research model shown to influence users’ satisfaction in a community-based telehealth program [27]. The model proposed by Hsieh *et al*. [27] provided a framework to synthesise the data for this systematic review given the paucity of literature that defines the construct of satisfaction with telehealth.

## Results

### Search results

**Fig 1:**
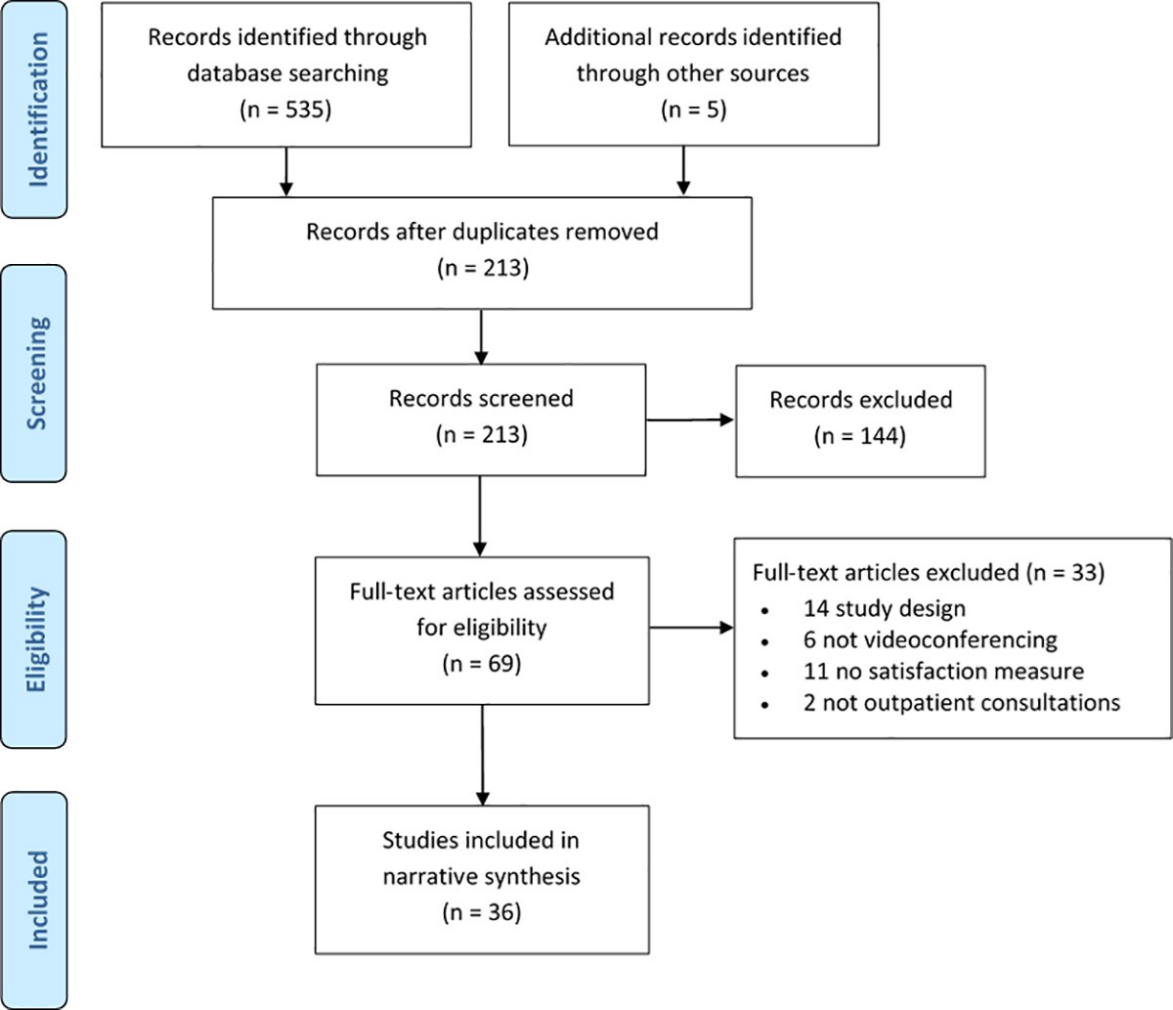
CONSORT diagram.

The initial search identified 535 studies. After pooling searches and removing duplicates, titles and abstracts were screened leaving 64 potentially relevant studies. Pearling through reference lists identified five additional studies. The full-texts were retrieved and assessed for eligibility and 36 studies were identified as being eligible for review. The literature selection process is outlined in Fig 1.

### Study characteristics

Table 1 provides an overview of the included studies. The included studies were published between 2003 and 2017. There were five clinical trials, 26 observational (cohort) studies and five qualitative studies. The majority of studies were conducted in the United States of America (n = 19), followed by Canada (n = 7) and Australia (n = 6). The cumulative number of participants across all studies totalled 3607. Thirty-four studies included patients while four studies set in paediatric or geriatric settings included the caregivers of patients.

**Table 1:** Summary of individual studies.

Author	Sample	Clinical description	Country	Outcome measure	Results
Medical Specialties					
AlAzab & Khader [52]	n = 64 patients	Nephrology: kidney disease	Jordan	Satisfaction questionnaire 15-items measured time and cost savings, scheduling, treatment effect, clinical management.	Greater than 90% of responses indicated satisfaction with telehealth.
Davis *et al*. [46]	n = 283 patients	Neurology: chronic neurological disorders	USA	Satisfaction questionnaire Items measured communication quality, quality of care, time and cost savings, convenience, confidentiality, overall satisfaction.	Greater than 90% of responses indicated satisfaction with telehealth. 95% wanted to continue their care via telehealth.
Hanlon-Dearman *et al*. [31]	n = 16 caregivers	Paediatrics: foetal alcohol spectrum disorder	Canada	Semi-structured interviews	Greater than 80% of respondents indicated satisfaction with telehealth. Themes related to caregiver satisfaction, included: convenience of accessing services locally especially with young children, good audio-visual quality, respect and sensitivity by clinicians, support of local clinicians/ coordinators in the telehealth session.
Mashru *et al*. [28]	n = 50 patients	Infectious diseases	Canada	Satisfaction questionnaire 9-items measured information comprehension, technical operation, audio-visual quality, confidentiality, overall satisfaction.	Greater than 90% of responses indicated satisfaction with telehealth.
Poulsen *et al*. [36]	n = 49 patients	Rheumatology	Australia	Satisfaction questionnaire 20-items measured information completeness and comprehension, evaluation credibility, clinician empathy and rapport, confidentiality, self-efficacy, patient preferences, time and cost savings, audio-visual quality, overall satisfaction.	Greater than 80% of responses indicated satisfaction with telehealth.
Powers *et al*. [48]	n = 13 patients n = 8 caregivers	Geriatrics: dementia	USA	Satisfaction questionnaire Items measured accessibility, technical operation, communication quality, overall satisfaction.	Greater than 80% of responses indicated satisfaction with telehealth.
Qiang & Marras [37]	n = 34 patients	Neurology: Parkinson’s disease	USA	Satisfaction questionnaire 11-items measured comfort, audio-visual quality, accessibility, communication quality, clinician empathy, confidentiality, overall satisfaction.	Greater than 80% of responses indicated satisfaction with telehealth.
Saifu *et al*. [49]	n = 30 patients	Hepatology: Human Immunodeficiency and Hepatitis C	USA	Satisfaction questionnaire 6-items measuring clinician rapport, confidentiality, physical environment, convenience, patient preferences, overall satisfaction.	Greater than 90% of responses indicated satisfaction with telehealth.
Saqui *et al*. [60]	n = 11 patients	Gastroenterology: parenteral nutrition	Canada	Satisfaction questionnaire 9-items measured communication quality, information informative, clinician empathy, patient expectations, shared decision making.	Greater than 80% of responses indicated satisfaction with telehealth. All patients were generally satisfied with videoconferencing as an alternative method of communication to face-to-face consultations.
Siminerio *et al*. [50]	n = 35 patients	Diabetes	USA	Satisfaction questionnaire 20-items measured overall satisfaction, information informative, evaluation credibility, communication quality, technology credibility, accessibility, convenience, confidentiality, shared decision making, time savings, self-efficacy.	Greater than 80% of responses indicated satisfaction with telehealth. 85% respondents agreed or strongly agreed with all items, evaluation credibility in absence of physical examination (66% satisfied), access to contact clinician via telehealth (83% satisfied).
Tokuda *et al*. [61]	n = 17 patients	Diabetes	USA	Focus groups	Satisfaction with videoconferencing was a key theme in all four focus groups. Participants expressed enjoyment of the videoconference visits and this was linked to participants’ engagement with health care and autonomy to manage their conditions. Satisfaction with information quality and cultural competency of clinicians towards rural patients were other identified themes.
**Rehabilitation**					
Burns *et al*. [29]	n = 38 patients	Speech Pathology	Australia	Satisfaction questionnaire 4-items measured technical operation, audio-visual quality.	Greater than 90% of responses indicated satisfaction with telehealth.
Grogan-Johnson *et al*. [30]	n = 29 patients n = 22 caregivers	Speech Pathology	USA	Satisfaction questionnaire 5-items for patients measured enjoyment, treatment effect, audio-visual quality. 7-items for caregivers measured technology use, communication quality, treatment effect, patient attitudes, patient preferences.	Greater than 80% of responses indicated satisfaction with telehealth.
Ramkumar *et al*. [38]	n = 87 caregivers	Audiology	India	Semi-structured interviews	Greater than 70% of responses indicated satisfaction with telehealth. Themes related to caregiver satisfaction included: equivalent to face-to-face, good audio-visual quality, new experience and ease of access to health care in a local community. Poor satisfaction was related to poor visual quality, inability to view the health professional, inability to ask questions of the health professional.
Schein *et al*. [41]	n = 48 patients	Wheeled mobility and seating	USA	Satisfaction questionnaire 7-items measured comfort, evaluation credibility, usability, audio-visual quality, time and cost savings.	Greater than 90% of responses indicated satisfaction with telehealth.
Ward *et al*. [42]	Rehabilitation n = 82 patients	Speech Pathology	Australia	Satisfaction questionnaire 14-items measured comfort, audio-visual quality, evaluation credibility, communication quality, information comprehension, time and cost savings, patient preferences.	Greater than 70% of responses indicated satisfaction with telehealth. Two-thirds of participants still preferred face-to-face consultations over telehealth.
**Genetic Counselling**					
Abrams & Geier [59]	n = 7 patients	Prenatal	USA	Satisfaction questionnaire Items measured usefulness, communication quality, confidentiality, overall satisfaction.	Greater than 80% of responses indicated satisfaction with telehealth.
Bradbury *et al*. [53]	n = 61 patients	Cancer	USA	Satisfaction questionnaire 13-items measured comfort, audio-visual quality, technical operation, communication quality, confidentiality, patient preferences, overall satisfaction.	Greater than 70% of responses indicated satisfaction with telehealth, except 52% reported technical difficulties with the telehealth technology.
Lea *et al*. [47]	n = 26 patients	Mixed	USA	Satisfaction numeric rating scale	Greater than 80% of responses indicated satisfaction with telehealth. Additional comments reported satisfaction with convenience of accessing telehealth, avoided travel and involvement of family members and local health professionals. Two patients still preferred to see the clinician in person.
Meropol *et al*. [56]	n = 31 patients	Cancer	USA	Satisfaction questionnaire 28-items measured usability, information informative, communication quality, patient comfort, patient preferences, overall satisfaction.	Greater than 80% of responses indicated satisfaction with telehealth.
Zilliacus *et al*. [45]	n = 12 patients	Cancer	Australia	Semi-structured interviews	Overall patients were highly satisfied with videoconferencing. Themes related to patient satisfaction included: audio-visual quality, convenience and reduced travel and associated costs, emotional support, clinician rapport. One participant woman with a recent cancer diagnosis, reported that telehealth was unable to meet her needs for psychosocial support.
**Oncology**					
Mooi *et al*. [33]	n = 11 patients	Mixed	Australia	Satisfaction questionnaire 16-items measured audio-visual quality, clinician empathy and rapport, confidentiality, information completeness and comprehension, communication quality, time and cost savings, patient preferences, overall satisfaction.	Greater than 80% of responses indicated satisfaction with telehealth.
Sabesan *et al*. [40]	n = 50 patients	Mixed	Australia	Satisfaction questionnaire 17-items measured communication quality, information completeness, evaluation credibility, clinician empathy and rapport, confidentiality, local clinical support, patient preferences, convenience, time and cost savings, audio-visual quality.	Greater than 80% of responses indicated satisfaction with telehealth. 76% of users thought it necessary to have the specialist complete a physical examination, although this occurred through a local doctor. 24% of users thought it necessary for a local doctor or nurse to accompany them during the videoconference. 82% preferred to received care through the videoconference than travel to the metropolitan.
Watanabe *et al*. [57]	n = 44 patients	Palliative Care and Radiotherapy	Canada	Satisfaction questionnaire 5-items measured staffing numbers, scheduling, overall satisfaction.	Greater than 80% of responses indicated satisfaction with telehealth. Three participants (6.8%) expressed discomfort with the telehealth equipment or format.
Weinerman *et al*. [44]	n = 34 patients	Gastrointestinal malignancy	Canada	Satisfaction questionnaire 13-items measured ability to communication quality, information completeness, clinician empathy, confidentiality, evaluation credibility, audio-visual quality, patient preferences, convenience.	Greater than 90% of responses indicated satisfaction with telehealth.
**Mental Health**					
Hassija & Gray [32]	n = 15 patients	Trauma counselling	USA	Satisfaction questionnaire 11-items measured audio-visual quality, technical operation, confidentiality, scheduling, patient expectations, clinician empathy, quality of care.	Greater than 90% of responses indicated satisfaction with telehealth.
Hilty *et al*. [63]	n = 67 patients	Psychiatry	USA	Satisfaction numeric rating scale	Greater than 90% of responses indicated satisfaction with telehealth.
Morland *et al*. [34]	n = 61 patients	Trauma counselling	USA	Satisfaction questionnaire 11-items measured audio-visual quality, comfort, usefulness, communication quality, evaluation credibility, service quality, patient expectations.	Greater than 80% of responses indicated satisfaction with telehealth.
Simpson *et al*. [42]	n = 6 patients	Psychotherapy: eating disorders	Scotland	Satisfaction questionnaire 4-items measured audio-visual quality, self-efficacy, overall satisfaction.	Greater than 70% of responses indicated satisfaction with telehealth. Additional comments reported satisfaction with convenience, anonymity of telehealth and comfort. Disadvantages were related to less personal, viewing self on screen.
**Primary Care**					
Mendez *et al*. [55]	n = patients unknown	General Practice	Canada	Satisfaction questionnaire Items measured comfort, technology usefulness.	Greater than 80% of responses indicated satisfaction with telehealth.
Polinski *et al*. [35]	n = 1734 patients	General Practice	USA	Satisfaction questionnaire 8-items measured patient understanding of telehealth, audio-visual quality, technical operation, quality of care, treatment quality, convenience and accessibility, overall satisfaction.	Greater than 90% of responses indicated satisfaction with telehealth. Predictors of liking telehealth were female gender (OR = 1.68, 1.04–2.72) and being very satisfied with their overall understanding of telehealth (OR = 2.76, 1.84–4.15), quality of care received (OR = 2.34, 1.42–3.87), and telehealth’s convenience (OR = 2.87, 1.09–7.94).
**Obstetrics & Gynaecology**					
Ferris *et al*. [62]	n = 263 patients	Gynaecology	USA	Satisfaction numeric rating scale	Greater than 90% of responses indicated satisfaction with telehealth.
Grindlay *et al*. [51]	n = 25 patients	Obstetrics	USA	Semi-structured interviews	Themes related to user satisfaction, included: convenience of accessing services locally, acceptability of conversing via technology, confidence in privacy and security. A small number preferred the anonymity of telehealth to discuss personal information. A small number preferred face-to-face consultations.
**Other**					
Friesner & Scott [58]	n = 96 patients	Pharmacy	USA	Satisfaction questionnaire 20-items measured communication quality, clinician empathy, professionalism, information informative, clinical management, confidentiality, overall satisfaction.	Greater than 90% of responses indicated satisfaction with telehealth. Items with the highest ratings were overall service quality and staff courtesy and respect.
López *et al*. [54]	n = 121 patients	Dermatology and General Surgery	Colombia	Satisfaction questionnaire 8-items measured overall satisfaction, communication quality, clinician empathy, comfort, usability.	Greater than 70% of responses indicated satisfaction with telehealth.
Roberts *et al*. [39]	n = 27 patients	Anaesthesia: pre-operative assessment	Australia	Satisfaction questionnaire 10-items measured audio-visual quality, time and cost savings, technology credibility, comfort, patient preferences.	Greater than 80% of responses indicated satisfaction with telehealth.

All studies utilised videoconferencing technologies to provide outpatient appointments between patients in their local health care centre and a health care provider at another centre. The clinical areas included medical specialties (n = 11), rehabilitation (n = 5), genetic counselling (n = 5), oncology (n = 4), mental health (n = 4), primary care (n = 2), obstetrics and gynaecology (n = 2), pharmacy (n = 1), dermatology and general surgery (n = 1) and anaesthetics (n = 1). Details of the videoconferencing technology were provided in two-thirds of studies. These studies used a monitor and camera linked to a computer that operated via an Integrated Services Digital Network. Few studies explicitly stated use of a dedicated broadband internet connection allowing data encryption. Additional digital equipment, such as handheld cameras, otoscopes and electronic stethoscopes were used by 10% of studies. The services were physician-led in almost half of the studies, followed by allied health (for example, genetic counsellor, psychologist, speech pathologist), nurse and multi-disciplinary teams. A nurse or telehealth technician was available onsite with the patient in one third of studies to assist with appointment scheduling, technology assistance or patient examination.

Patient and caregivers’ satisfaction with telehealth were measured using a range of different methods such as questionnaires (n = 28), numerical rating scores (n = 3), semi-structured interviews (n = 4) and focus groups (n = 1). The measures used were often developed for each study’s unique setting, resulting in heterogeneity of measures.

### Methodological quality assessment

The results of the assessment of methodological quality are outlined in Tables 2 and 3. There was significant variation in the methodological quality scores. Of the 31 quantitative studies, 15 scored greater than 70% while 16 scored below 70%. Amongst the quantitative research designs, studies were scored lower due to inadequate reporting of outcome measure reliability and validity, lack of justification of sample size, drop-outs and inappropriate statistical methods. Amongst the five studies using qualitative research methodologies, three scored great than 70% while two scored below 70%. Studies scored lower due to small sample sizes, inadequate descriptions of data collection procedures and lack of rigour in the data analysis process.

**Table 2:** Methodological quality of quantitative studies based on the McMaster critical appraisal tool.

**Citations**	**Items**														**Score**	
	Purpose	Literature review	Sample description	Sample size	Outcome reliability	Outcome validity	Intervention described	Contamination	Co-interventions avoided	Statistical significance	Analysis appropriate	Clinical significance	Dropouts reported	Conclusions appropriate	Raw	%
**Clinical trials**																
**Ferris *et al*. [62]**	Y	Y	Y	N	N	N	Y	n/a	n/a	Y	Y	Y	N	Y	8	67
**Grogan-Johnson *et al*. [30]**	Y	Y	Y	N	N	N	Y	n/a	n/a	N	Y	Y	Y	Y	8	67
**Hilty *et al*. [63]**	Y	N	Y	N	N	N	Y	n/a	n/a	Y	Y	N	N	Y	5	42
**Morland *et al*. [34]**	Y	Y	Y	Y	N	N	Y	n/a	n/a	Y	Y	Y	Y	Y	10	83
**Weinerman *et al*. [44]**	Y	Y	Y	Y	N	N	Y	n/a	n/a	Y	Y	Y	Y	Y	10	83
**Cohorts**																
**Abrams & Geier [59]**	Y	Y	N	N	N	N	Y	n/a	n/a	N	Y	N	Y	Y	6	50
**AlAzab & Khader [52]**	Y	Y	Y	Y	N	N	Y	n/a	n/a	Y	Y	Y	Y	Y	10	83
**Bradbury *et al*. [53]**	Y	Y	Y	Y	N	N	Y	n/a	n/a	N	Y	Y	Y	Y	9	75
**Burns *et al*. [29]**	Y	N	Y	N	N	N	Y	n/a	n/a	N	Y	Y	N	Y	6	50
**Davis *et al*. [45]**	Y	Y	Y	N	N	N	Y	n/a	n/a	N	Y	Y	N	Y	7	58
**Friesner & Scott [58]**	Y	Y	Y	Y	Y	Y	Y	n/a	n/a	Y	Y	Y	Y	Y	12	100
**Hassija & Gray [32]**	Y	Y	Y	N	N	N	Y	n/a	n/a	Y	Y	Y	Y	Y	9	75
**Lea *et al*. [47]**	Y	Y	Y	N	N	N	Y	n/a	n/a	N	Y	Y	Y	Y	8	67
**López *et al*. [54]**	Y	N	Y	N	N	Y	Y	n/a	n/a	N	Y	Y	Y	Y	8	67
**Mashru *et al*. [28]**	Y	Y	Y	N	N	N	Y	n/a	n/a	N	Y	N	N	Y	6	50
**Mendez *et al*. [55]**	Y	N	N	N	N	N	Y	n/a	n/a	N	N	Y	N	Y	4	33
**Meropol *et al*. [56]**	Y	Y	Y	N	N	N	Y	n/a	n/a	N	Y	Y	N	Y	7	58
**Mooi *et al*. [33]**	Y	Y	Y	N	N	N	Y	n/a	n/a	N	Y	Y	Y	Y	8	67
**Polinski *et al*. [35]**	Y	Y	Y	N	N	N	Y	n/a	n/a	Y	Y	Y	Y	Y	9	75
**Poulsen *et al*. [36]**	Y	Y	Y	N	N	N	Y	n/a	n/a	Y	Y	Y	Y	Y	9	75
**Powers *et al*. [48]**	Y	Y	Y	N	N	N	Y	n/a	n/a	N	Y	Y	Y	Y	8	67
**Qiang & Marras [37]**	Y	Y	Y	N	N	N	Y	n/a	n/a	Y	Y	Y	Y	Y	9	75
**Roberts *et al*. [39]**	Y	Y	Y	N	N	Y	Y	n/a	n/a	Y	Y	Y	Y	Y	10	83
**Sabesan *et al*. [40]**	Y	Y	Y	N	N	Y	Y	n/a	n/a	N	Y	Y	Y	Y	9	75
**Saifu *et al*. [49]**	Y	N	Y	N	N	N	Y	n/a	n/a	Y	Y	Y	Y	Y	7	58
**Saqui *et al*. [60]**	Y	N	Y	N	N	N	Y	n/a	n/a	N	Y	N	Y	Y	6	50
**Schein *et al*. [41]**	Y	Y	Y	Y	N	N	Y	n/a	n/a	Y	Y	Y	Y	Y	10	83
**Siminerio *et al*. [50]**	Y	Y	Y	N	N	N	Y	n/a	n/a	Y	Y	Y	Y	Y	9	75
**Simpson *et al*. [42]**	Y	Y	Y	N	N	N	Y	n/a	n/a	N	Y	Y	N	Y	7	58
**Ward *et al*. [43]**	Y	Y	Y	Y	N	N	Y	n/a	n/a	Y	Y	Y	Y	Y	10	83
**Watanabe *et al*. [57]**	Y	Y	Y	N	N	N	Y	n/a	n/a	Y	Y	Y	Y	Y	9	75

Y = yes (green shading); N = no (red shading); n/a = not applicable (grey shading).

**Table 3:** Methodological quality of qualitative studies based on the McMaster critical appraisal tool.

**Citations**	**Items**																						**Score**	
	Purpose	Literature review	Theoretical perspective	Sample described	Sampling redundancy	Informed consent	Site described	Participants described	Researcher relationship described	Researcher biases described	Procedural rigor	Data analysis	Findings consistent	Auditable trail	Data analysis	Theoretical connections	Credible	Transferable	Dependable	Confirmable	Conclusions appropriate	Clinical implications	Raw	%
**Interviews**																								
**Grindlay *et al*. [51]**	Y	Y	Y	Y	N	Y	Y	Y	N	N	N	Y	Y	N	N	Y	Y	Y	Y	N	Y	Y	15	68
**Hanlon-Dearman *et al*. [31]**	Y	Y	Y	Y	Y	Y	Y	Y	Y	Y	Y	Y	Y	Y	Y	Y	Y	Y	Y	Y	Y	Y	22	100
**Ramkumar *et al*. [38]**	Y	Y	Y	Y	Y	Y	Y	Y	N	Y	Y	Y	Y	N	Y	Y	Y	Y	Y	Y	Y	Y	20	91
**Zilliacus *et al*. [45]**	Y	Y	Y	Y	N	Y	Y	Y	N	Y	Y	Y	Y	N	Y	Y	Y	Y	Y	Y	Y	Y	19	86
**Focus group**																								
**Tokuda *et al*. [61]**	Y	Y	Y	Y	N	Y	Y	N	N	N	N	N	N	N	N	N	Y	Y	Y	N	Y	Y	11	50

Y = yes (green shading); N = no (red shading).

### System experience

System experience was the most commonly measured dimension of satisfaction appearing in 29 studies (81%). It included the audio-visual quality of videoconferencing [28–45], accessibility of a service in one’s local health care centre [31, 35, 37, 38, 40, 44–51], time and cost savings for patients [33, 36, 39–41, 43, 45, 46, 50, 52], patient comfort in participating in telehealth [34, 37, 39, 41–43, 53–57], technical support and operations [28, 29, 31, 32, 35, 48, 53] and usability of telehealth technology [41, 54, 56]. There were high levels of satisfaction across all these domains, especially with regards to service accessibility. This was linked to convenience of attending an appointment in one’s local community, saving travel time and costs [31, 35, 36, 42, 47, 51].

There were minor accounts of discomfort with screen formatting and using equipment [42, 53, 57]. Reduced audio-visual quality also affected satisfaction and patient and caregivers’ ability to participate in the appointment [38].

### Information sharing

Satisfaction with information sharing was measured by 21 studies (58%). It included the communication quality between the patient and health care provider [30, 33, 34, 37, 40, 43, 44, 46, 48, 50, 53, 54, 56, 58, 59, 60], patient confidentiality [28, 32, 33, 36, 37, 40, 44, 46, 49, 50, 53, 58, 59], thoroughness of clinical assessment [34, 36, 40, 41, 43, 44, 50], information completeness [33, 36, 40, 44] and usefulness [50, 56, 58, 60] and patient comprehension [28, 33, 36, 40, 43]. There were high levels of satisfaction across all these domains.

Communication between the patient and health care provider positively influenced satisfaction with telehealth by enabling patients to feel listened to, have their concerns addressed, have time to ask questions and participate in the information sharing and decision making. Patients were mostly satisfied that information was safely shared and remained confidential, though there were minor reports that videoconferencing might increase the risk of breach of confidentiality of patients’ health information [53]. Two studies reported that the anonymity of telehealth was helpful in discussing personal information that were perceived to be more difficult to discuss face-to-face [42, 51]. In absence of physical examination, some patients remained neutral or less satisfied with telehealth reporting 33–75% dissatisfaction [36, 40, 50]. One study found patients preferred to have their local doctor or nurse present during the telehealth appointment [33].

### Consumer focus

Satisfaction with consumer focus was measured by 17 studies (47%). It included adherence to patient-centred care [30, 32–36, 39, 40, 42–44, 49–51, 53, 56, 60, 61], health care provider empathy and rapport with the patient [31–33, 36, 37, 40, 44, 49, 54, 58, 60], quality of care [32, 35, 46], local health care provider support [40], emotional support [45], professionalism [58] and health care provider cultural competence [61]. Patients and caregivers were satisfied across all these domains of consumer focus with telehealth.

Patient centred-care was a large domain within consumer focus. It measured services’ responsiveness to patient preferences, needs and values. While face-to-face appointments were preferred, telehealth remained a satisfactory option through avoided travel and costs from attending an appointment in an urban centre [36, 39, 40, 43]. The studies were unclear why face-to-face appointments were preferred in these settings but hypothesised an older demographic [43], the perceived need to develop rapport with the health care provider [36] and unfamiliarity with telehealth [40] as factors potentially influencing patient choices. There were accounts of improved self-efficacy in managing one’s condition via telehealth [36, 42, 50], enjoyment of the telehealth experience [30, 61], positive attitudes of receiving health care via telehealth [30] and agreement that patient treatment needs were being met [32, 34, 60]. There were only minor reports of telehealth being less personal [42] and unable to provide psychosocial support [45].

### Overall satisfaction

Overall satisfaction was measured by 24 studies (67%). This gross measure was measured in a number of ways, including on a numeric rating scale, as a specific question within a satisfaction questionnaire and qualitatively through semi-structured interviews. Numeric rating scale was used by three studies [47, 62, 63] ranging from 4.45 to 4.7 out of 5. Respondents of questionnaires scored greater than 80% agreement in overall satisfaction [28, 33, 35–37, 42, 46, 48–50, 53, 54, 56–59]. In the semi-structured interviews, 81% of participants reported being satisfied with the telehealth experience [31].

### Summary of results

Summarising the findings from quantitative and qualitative research, there is consistent evidence that telehealth has an overall positive impact on patient and caregivers’ satisfaction. System experience seems to enhance better access to health care for patients and their caregivers living in rural and remote areas highlighting that distance may no longer be a barrier. Telehealth also appears to enhance communication and engagement between health care providers and patients and their caregivers, especially through real-time videoconferencing. Irrespective of information and communication technologies, consumer focus remained a critical aspect of how a service was delivered. Consumer focus should not be compromised with the advances in information and communication technologies; but rather improve the functional delivery of telehealth.

## Discussion

While telehealth has been promoted as a viable and alternative mode of service delivery, especially for people living in rural and remote areas to access health care within their local communities, to date there has been limited quality research to evaluate and report on patient and caregivers’ satisfaction with telehealth. This systematic review aimed to address this knowledge gap, with a particular focus on videoconferencing, and identified a large body of evidence consisting of 36 studies of varying designs from a range of clinical settings. The summarised findings indicate that patients and caregivers were indeed satisfied with telehealth due to a range of reasons. Specifically, attending an appointment in one’s local community via telehealth outweighed the inconvenience of travelling long distances to an urban centre for the same appointment. This was especially highlighted for people with chronic conditions [36, 41, 46, 50], parents with young children [30, 31, 38] and caregivers of elderly patients [48]. As this systematic review had a focus on videoconferencing, these findings are limited to this modality of telehealth and cannot be generalised to other modalities of telehealth.

Technology plays a crucial role in addressing barriers to health care access for people living in rural and remote areas. Videoconferencing enables real-time audio-visual communication with outcomes not significantly different from face-to-face appointments [13]. Informed by this view, there is an increasing body of research examining the impact of home-based telehealth [56, 64–67]. Advances in technology and improved consumer internet connectivity and computer literacy may empower people in rural and remote areas to manage their health conditions by better connecting to health care services [68, 69].

Despite advances in technology, a number of challenges are known to influence the success and sustainability of telehealth in rural and remote areas. Governance and stakeholder support; demonstrated economic value with consistent activity reimbursement capacity; service adaptability to the targeted population; and efficient administrative and clinical processes are some of the known challenges to using telehealth [70, 71]. In addition, not all jurisdictions may be appropriately resourced with infrastructure technology. Whilst the case for telehealth from the patient’s perspective is strengthened from this review, most of the studies were set in high-income countries with few from low-income countries [38, 52, 66]. One study set in India was influenced by an interrupting satellite signal and inconsistent audio-visual quality [38]. Poor infrastructure technology in these settings, in addition to higher running costs and low technical expertise present limitations for telehealth delivery and access [11, 72].

While technology undoubtedly is critical and is a valuable tool to address the tyranny of distance, importantly it should be complemented with the functional aspects of service delivery. Health care provider communication and empathy are universal to health care and relate directly to patient and caregivers’ satisfaction [73–76]. The findings from this review reinforce the need for health care providers to actively engage and partner with patients when face-to-face appointments have been substituted for videoconferencing. Telehealth can still remain a personal experience through elements of the communication skills that include listening to patients, providing adequate time for patient questioning, investing time in building patient rapport, involving caregivers in the appointment and emphasising patient choice. How well the telehealth experience aligned with patient values and expectations may causally influence their satisfaction as well as health care outcomes.

This systematic review demonstrates the positive impact on patient and caregivers’ satisfaction with telehealth and builds on the findings from previous systematic reviews [20, 21]. This review grouped patient satisfaction with telehealth into four dimensions: system experience, information sharing, consumer focus and overall satisfaction. While a true construct of satisfaction remains debateable from a methodological point of view [77], the findings from this review add to the current knowledge base on satisfaction being a multi-dimensional construct.

### Limitations

As with any research, there are limitations to this systematic review. While the systematic searching of the literature identified a large body of evidence to inform the review topic, there were concerns regarding the methodological quality of the included studies. The areas of concern include small sample sizes and heterogeneity in terms of how satisfaction was defined and measured, at times using psychometrically untested instruments. In some studies, satisfaction was not the primary outcome of interest but rather a secondary measure resulting in limited reporting of relevant data. While this systematic review process was underpinned by best practice in the conduct of systematic reviews (PRISMA), likely publication and language bias should be acknowledged. While strategies were implemented to avoid publication bias, such as grey literature and secondary searching, due to the complexity and imprecise nature of searching and identifying grey literature, especially when investigating a complex and undefined concept such as satisfaction, some publications may have been missed.

### Implications for practice

There is a large body of evidence to indicate that patients and caregivers are generally satisfied with telehealth. This complements the widely held view that telehealth can play an important role for supporting rural and remote patients where clinically appropriate and hence avoid the inconvenience of travel to an urban centre for a face-to-face appointment. While health care providers should not underestimate patients’ ability to engage with technology, this could be complemented with functional aspects of care, as they would in a face-to-face appointment. Given patient and caregivers’ satisfaction with telehealth, health services could feel confident that this form of service delivery enables health care for patients in rural and remote areas and is not a barrier from the patient and/or caregivers’ perspective. While telehealth does not replace face-to-face appointments, it does offer an alternative mode of service delivery that when integrated into an established service could form part of patient choice when clinically safe and appropriate. Aligning a health care service with patients’ expectations and needs can lead to overall patient satisfaction.

### Implications for research

Growing evidence supports the use of telehealth from both the patient and caregivers’ perspectives. However methodological concerns of the current evidence may guide the direction of future research. Future research should address the question of how to accurately measure satisfaction given this is a routinely used measure in health service. Similarly, future research may also improve the current evidence base by reporting on follow-up research, which investigate if patient and caregivers’ satisfaction with telehealth can be sustained over the long term.

## Supporting information

S1 File PRISMA checklist. (DOC)

S2 File Appendix. (DOCX)
