# Supplementary material for: Systematic review of patient and caregivers’ satisfaction with telehealth videoconferencing as a mode of service delivery in managing patients’ health
Source: PLoS One. 2019 Aug 30;14(8):e0221848. doi: 10.1371/journal.pone.0221848 (PMC6716655; doi:10.1371/journal.pone.0221848)
Supplement: S2 File — (DOCX) [file pone.0221848.s002.docx]

**Appendix**

**PICOT Table**

| **Population** | Patients and/or their caregivers  Living in inner regional, outer regional, remote or very remote areas  Any age  Any health condition |
| --- | --- |
| **Intervention** | Outpatient appointment delivered remotely via telehealth videoconferencing between the patient in their home or local health care centre and the health care provider in another location |
| **Comparator** | No comparator |
| **Outcome** | Patient and/or caregiver satisfaction with telehealth videoconferencing  Qualitative or quantitative data |
| **Time** | No timeframe specified |

**Search strategy**

Incorporated into electronic databases (Ovid Medline, Embase, CINAHL, ProQuest Health Research Premium Collection, Joanna Briggs Institute and the Cochrane Library).

| **Search number** | **Search terms** |
| --- | --- |
| 1 | telehealth.mp. |
| 2 | exp Telemedicine/ |
| 3 | exp teleconsult/ |
| 4 | exp Remote Consultation/ |
| 5 | exp Videoconferencing/ |
| 6 | 1 or 2 or 3 or 4 or 5 |
| 7 | exp Patient Satisfaction/ |
| 8 | exp Caregivers/ |
| 9 | 7 or 8 |
| 10 | exp Rural Health/ |
| 11 | exp Rural Population/ |
| 12 | exp Rural Health Services/ |
| 13 | 10 or 11 or 12 |
| 14 | 6 AND 9 AND 13 |
